# Interleukin-4 regulates lipid metabolism by inhibiting adipogenesis and promoting lipolysis

**DOI:** 10.1194/jlr.M041392

**Published:** 2014-03

**Authors:** Chang-Hui Tsao, Ming-Yuh Shiau, Pei-Hua Chuang, Yih-Hsin Chang, Jaulang Hwang

**Affiliations:** * Graduate Institute of Life Sciences, National Defense Medical Center, Taipei, Taiwan; † Department of Biotechnology and Laboratory Science in Medicine, National Yang-Ming University, Taipei, Taiwan; § Department of Nursing, College of Medicine and Nursing, Hungkuang University, Taichung, Taiwan; ** Department of Biochemistry, School of Medicine, Taipei Medical University, Taipei, Taiwan

**Keywords:** peroxisome proliferator-activated receptor-γ, CCAAT/enhancer-binding protein-α, hormone sensitive lipase, perilipin

## Abstract

Long-term cytokine-mediated inflammation is a risk factor for obesity and type 2 diabetes mellitus (T2DM). Our previous studies reveal significant associations between promoter single nucleotide polymorphisms (SNPs) of interleukin (IL)-4 and T2DM, as well as between SNPs in genes encoding IL-4/IL-4 receptor and high density lipoproteins. Our animal study reveals that IL-4 regulates glucose/lipid metabolism by promoting glucose tolerance and inhibiting lipid deposits. The above results strongly suggest the involvement of IL-4 in energy homeostasis. In the present study, we focus on examining the regulatory mechanism of IL-4 to lipid metabolism. Our results show that IL-4 inhibits adipogenesis by downregulating the expression of peroxisome proliferator-activated receptor-γ and CCAAT/enhancer-binding protein-α. Additionally, IL-4 promotes lipolysis by enhancing the activity and translocation of hormone sensitive lipase (HSL) in mature adipocytes, which suggests that IL-4 plays a pro-lipolytic role in lipid metabolism by boosting HSL activity. Our results demonstrate that IL-4 harbors pro-lipolysis capacity by inhibiting adipocyte differentiation and lipid accumulation as well as by promoting lipolysis in mature adipocytes to decrease lipid deposits. The above findings uncover the novel roles of IL-4 in lipid metabolism and provide new insights into the interactions among cytokine/immune responses, insulin sensitivity, and metabolism.

Obesity is characterized by an expansion of white adipose tissue (WAT) mass resulting from increased adipocyte number and/or size (1). It is a key risk factor leading to type 2 diabetes mellitus (T2DM) and hyperlipidemia, and has become a pan-endemic health problem with rapidly growing global incidence (2, 3).

Obesity is associated with systemic chronic inflammation characterized by altered cytokine production and activation of inflammatory signaling (4, 5). Abundant studies have linked the increased production of inflammatory cytokines, such as tumor necrosis factor-α (TNF-α), interleukin (IL)-6, and certain adipokines, during the inflammatory process to obesity, as well as to the development of insulin resistance (6–8). However, the effect of anti-inflammatory cytokines, such as IL-4, in the development of insulin resistance or obesity is less understood.

IL-4, secreted by activated Th2 lymphocytes, basophils, and mast cells, executes pleiotropic functions, such as induction of Th2 differentiation, immunoglobulin class switching, and B cell proliferation (9). The production of IL-4 by splenic lymphocytes from diet-induced obese mice is increased (10) and the amount of serum IL-4 is reduced in Sprague-Dawley rats after receiving visceral fat removal surgery (11). IL-4, secreted from adipocytes and hepatocytes, shows the capacity of modulating local immune response and insulin sensitivity (12, 13). These results suggest IL-4 may participate in the processes of diet-induced obesity and metabolism. In support of the abovementioned studies, significant associations between IL-4 genotypes and T2DM, as well as between IL-4 genotypes and circulatory levels of high density lipoprotein-cholesterol (HDL-C), are identified in our previous study (14). Our most recent report also demonstrates that polymorphisms of the IL-4 receptor gene are significantly associated with HDL-C (15). In addition, results from our animal study reveal that IL-4 improves insulin sensitivity and glucose tolerance while inhibiting lipid accumulation in fat tissues, which leads to decreased weight gain and fat mass (16). Taken together, these studies suggest that IL-4 may participate in lipid metabolism and diabetic susceptibility.

To further explore the roles of IL-4 in lipid metabolism, the pathogenesis of obesity, and T2DM, the present study aimed to elucidate the effects of IL-4 on adipogenesis and lipid metabolism by using adipocytes as a study model. Our data shows that IL-4 not only inhibits adipogenesis at the early phase of adipocyte differentiation through the signal transducer and activator of transcription 6 (STAT6) signaling pathway, but also promotes lipolysis by upregulating the activity of hormone sensitive lipase (HSL), the key enzyme for triacylglyceride degradation.

## MATERIALS AND METHODS

### Reagents

Mouse recombinant IL-4 was purchased from Millipore (Temecula, CA). Tyrphostin AG 490 (AG490) and ECL reagent were purchased from Calbiochem (Merck Millipore, Billerica, MA). 3-isobutyl-methylxanthine (IBMX), dexamethasone (Dex), insulin, isoproterenol (ISO), thiazolidinedione (TZD), and the free glycerol determination kit were purchased from Sigma (St. Louis, MO). Protein A/G beads, TRIzol reagent, and Applied Biosystems SYBR Green Realtime PCR Master Mix were purchased from Life Technologies (Carlsbad, CA). Small interfering RNA (siRNA) and DharmaFECT 1 reagent were purchased form Dharmacon (Lafayette, CO)

### Cell culture, adipogenesis of 3T3-L1 cells, and cell counting

3T3-L1 preadipocytes were maintained in DMEM containing 10% calf serum (Hyclone Laboratories, South Logan, UT). Two day postconfluent 3T3-L1 cells (designated day 0) were induced to differentiate by addition of a standard cocktail composed of 0.5 mM IBMX, 1 μM Dex, and 10 μg/ml insulin in 10% FBS for 2 days (designated as MDI cocktail). The cells were then cultured in DMEM supplemented with 10% FBS and 5 μg/ml insulin. The medium was replaced with fresh medium every 2 days. For the AG490 experiments, cells were preincubated with 10 μM AG490 for 1 h and then treated with 10 ng/ml IL-4. For cell counting, cells were harvested and stained with trypan blue (0.5%; Biological Industries, Kibbutz Beit Haemek, Israel), and viable cells were counted with the use of a hemocytometer (Lauda-Königshofen, Marienfeld-Superior, Germany).

### Oil Red O staining

Oil Red O (ORO) staining was performed as previously described (17). For quantification, the dye was eluted by adding 100% isopropanol and the extracts were determined by measuring the absorbance at 490 nm.

### Western blot analysis, immunodepletion, and immunoprecipitation assay

Cell lysates were prepared in RIPA buffer containing protease inhibitors, as described previously (18). Sixty micrograms of cell extracts were subjected to SDS-PAGE, transferred to a polyvinylidene difluoride membrane, and blotted with specific primary antibodies. Antibodies against CCAAT/enhancer binding protein (C/EBP)β, C/EBPδ, C/EBPα, PPARγ, fatty acid binding protein 4 (aP2) (FABP4), HSL, phospho-Ser^563^ HSL, perilipin, phospho-(Ser/Thr) protein kinase A (PKA) substrate, adipocyte triglyceride lipase (ATGL), and β-actin were purchased form Cell Signaling Technology (Danvers, MA). Anti-p27 Kip1 was purchased from GenTex, Inc. (Irvine, CA). Anti-STAT6/anti-phospho-STAT6 (Y641) and anti-IL-4 receptor α chain (IL-4Rα) were purchased from BD Biosciences (San Jose, CA) and R&D Systems (Minneapolis, MN), respectively. For detection, membranes were incubated with secondary antibodies (Merck Millipore) for 1 h, and results were visualized with ECL reagent and exposed to X-films. The blot was quantified by Labscan software. For immunodepletion assay, 250 μg cell lysates were incubated with 1 μg anti-perilipin antibody overnight and captured by protein A/G agarose for 2 h. Supernatant was harvested and subjected to Western blotting. For immunoprecipitation assay, 500 μg cell lysates were incubated with 1 μg anti-phospho-PKA substrate antibody overnight and captured by protein A/G agarose for 2 h. After washing with RIPA buffer, immunoprecipitates were eluted in sample buffer, denatured, and subjected to Western blotting as described.

### Real-time quantitative PCR

Total RNA was isolated by TRIzol reagent according to the manufacturer's instructions. About 2 μg of total RNA was reversed transcribed with the Improm-II reverse transcription kit (Promega, Madison, WI), and real-time PCR was performed using Applied Biosystems SYBR Green Realtime PCR Master Mix and the StepOnePlus™ real-time PCR system. The following primers were used for detection of the gene expression: C/EBPβ (forward, 5′-AAGCTGAGCGACGAGTACAAGA-3′; reverse, 5′-GTCAGCTCCAGCACCTTGTG); C/EBPδ (forward, 5′-TTCAGCGCCTACATTGACTC-3′; reverse, 5′-GCTTTGTGGTTGCTGTTGAAG-3′); PPARγ2 (forward, 5′-GATGCACTGCCTATGAGCACTT-3′; reverse-5′-AGAGGTCCACAGAGCTGATTCC-3′); C/EBPα (forward, 5′- AGCAACGAGTACCGGGTACG-3′; reverse, 5′-TGTTTGGCTTTATCTCGGCTC-3′); aP2 (FABP4) (forward, 5′-CACCGCAGACGACAGGAAG-3′; reverse, 5′-GCACCTGCACCAGGGC-3′); glucose transporter 4 (GLUT4) (forward, 5′-ACTCATTCTTGGACGGTTCCTC-3′; reverse, 5′-CACCCCGAAGATGAGTGGG-3′); and 18s rRNA (forward, 5′-CGGCTACCACATCCAAGGAA-3′; reverse, 5′-GCTGGAATTACCGCGGCT). All real-time PCR was done with following conditions: 10 min at 95°C, 40 cycles of 15 s at 95°C, and 30 s at 60°C. For semi-quantification RT-PCR, PCRs were optimized to determine the linear phase of amplification. Primer sequences used for amplification were FAS (forward, 5′-TGCTCCCAGCTGCAGGC; reverse, 5′-GCCCGGTAGCTCTGGGTGTA); LPL (forward, 5′-ATGGAGAGCAAAGCCCTGC; reverse, 5′-AGTCCTCTCTCTGCAATCAC); resistin (forward, 5′-AGACTGCTGTGCCTTCTGGG; reverse, 5′-CCCTCCTTTTCCTTTTCTTCCTTG); and adiponectin (forward, 5′-TCCTGGAGAGAAGGGAGAGAAAG; reverse, 5′-TCAGCTCCTGTCATTCCAACAT). Results are representative of three independent experiments.

### siRNA

For siRNA transfection, the 2 day postconfluent cells were transfected with 100 nM STAT6 siRNA (catalog number L-040690-01) or control siRNA (catalog number D-001810-10-05) by DharmaFECT 1 reagent according to the manufacturer's instructions. The cells were incubated in medium containing the siRNA-DharmaconFECT1 complex for 16 h. The medium was then replaced with DMEM supplemented with 10% calf serum, and the cells were incubated for an additional 24 h and then induced into differentiation.

### Electrophoretic mobility shift assay

Electrophoretic mobility shift assay (EMSA) was performed using the LightShift chemiluminescent EMSA kit (Thermo Scientific, Rockford, IL) according to the manufacturer's instructions. The postconfluent cells were induced by MDI for 24 h or 48 h and then nuclear extracts were collected for EMSA experiments by nucleocytoplasmic separation using a nuclear protein extraction kit (Thermo Scientific). The DNA sequence used for EMSA was 5′-GTATTTCCCAGAAAAGGAAC-3′. The probe was prepared by annealing oligonucleotides with their 3′-end labeled with biotin.

### Measurement of lipolysis

3T3-L1 cells were incubated in serum-free DMEM in the presence or absence of IL-4 and/or ISO. The medium was then analyzed for glycerol and nonesterified free fatty acids, respectively, with a free glycerol determination kit and free fatty acid kit (Biovision, Milpitas, CA).

### Confocal microscopy

Cells were fixed with 3.7% formaldehyde for 15 min and then permeabilized with 0.5% Triton X-100 for 15 min at room temperature. The cells were then blocked with 5% BSA for 30 min at room temperature followed by incubation with the primary antibody against phospho-Ser^563^ HSL overnight at 4°C. Goat anti-rabbit IgG-conjugated DyLight^TM^ 594 (Jackson ImmunoResearch Laboratories, West Grove, PA) was added together with BODIPY (Molecular Probes, Eugene, OR) and incubated for 1 h at room temperature. Cells were then mounted with Gel/Mount containing DAPI (Molecular Probes). The images were taken by using the Zeiss LSM 700 confocal fluorescence microscope system with a 63× objective lens.

### Statistical analysis

All values are presented as mean ± SEM. For statistical analysis, the *P* value was calculated using a two-tailed unpaired Student's *t*-test with *P* < 0.05 considered as statistically significant.

## RESULTS

### IL-4 inhibits adipogenesis

**Fig. 1.**
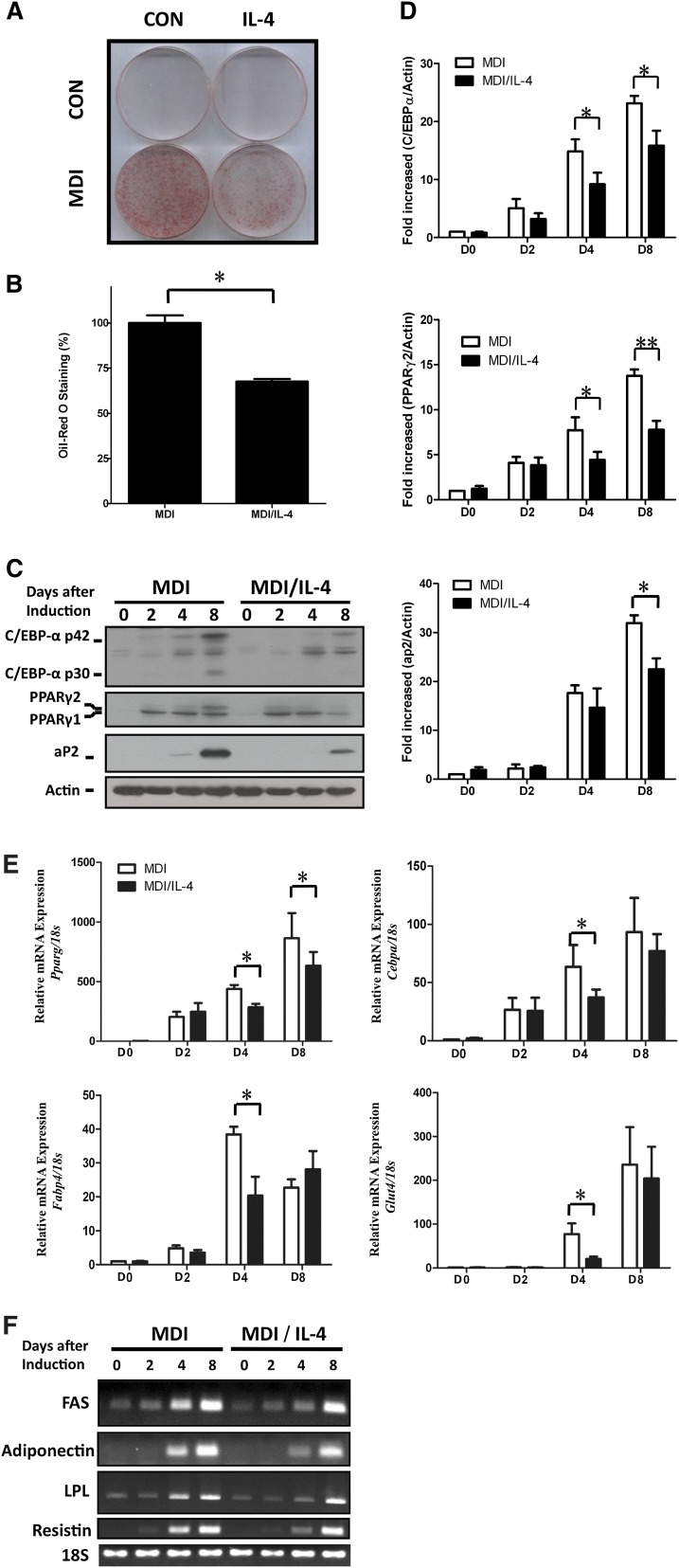
Effects of IL-4 on adipogenesis of 3T3-L1 cells. Lipid accumulation and expression levels of adipocyte-specific genes during adipogenesis under IL-4 treatment were analyzed. A: 3T3-L1 cells were induced into mature adipocytes in the absence or presence of 10 ng/ml of IL-4 and subjected to ORO staining. B: Quantification of the ORO staining results by measuring the corresponding absorbance of isopropanol-extracted dye at 490 nm. C: Protein expression of aP2, C/EBPα, C/EBPβ, and PPARγ in 3T3-L1 was analyzed at the indicated time. β-actin served as a loading control. D: Quantification of Western blotting by densitometer. The data are presented as the mean ± SEM (n = 7). *P < 0.05 and **P < 0.01 compared with MDI. E, F: mRNA expression of the aP2, C/EBPα (*Cebpa*), PPARγ (*Pparg*), GLUT4 (*Glut4*), and adipocyte-specific genes (adiponectin, *Adipoq*; resistin, *Retn*; fatty acid synthase, *Fasn*; lipoprotein lipase, *Lpl*) in 3T3-L1 was analyzed by real-time PCR (E) and RT-PCR (F), respectively, at the indicated time. All results were normalized to 18s rRNA. The data are presented as the mean ± SEM (n = 3), and statistically analyzed by two-tailed unpaired Student's *t*-test. *P < 0.05 compared with MDI. CON, control; D, day.

To evaluate the putative effects of IL-4 on adipogenesis, 3T3-L1 preadipocytes (19) were allowed to differentiate into mature adipocytes in the presence or absence of IL-4 during the entire differentiation period. The extent of the differentiation was evaluated by ORO staining. In addition, the expression of important genes mediating adipogenesis, such as C/EBPα and PPARγ, and makers of mature adipocytes, including aP2 and GLUT4, were analyzed. Results of ORO staining showed that lipid accumulation in differentiated cells was inhibited by ∼30% in the presence of IL-4 treatment (**Fig. 1A, B**). The expression of two important regulators for terminal adipogenesis, PPARγ and C/EBPα, were subsequently examined to explore the underlying inhibitory mechanism of IL-4 to adipogenesis. Figure 1C shows that IL-4 not only caused reduced levels, but also caused an expression lag of PPARγ and C/EBPα during the differentiation process (Fig. 1C, D). Meanwhile, expression of aP2, an adipogenic gene encoding a fatty acid-binding protein in mature adipocytes, was also significantly downregulated (Fig. 1C, D). The mRNA expression of *Pparg* and *Cebpa* was further analyzed by probing the regulatory mechanism of IL-4 treatment on adipogenesis. Consistent with the previous observations, *Pparg* and *Cebpa* mRNA expression was also reduced under IL-4 treatment during the entire differentiation process (Fig. 1E). The above observations indicate that IL-4 harbors inhibitory capacity to adipogenesis by suppressing the expression of important transcription factors driving adipocyte differentiation. Moreover, mRNA expression levels of adipokines, such as *Adipoq* and *Retn*, were temporally and significantly inhibited by IL-4 (Fig. 1F). The IL-4 inhibitory effects were also observed on genes involved in glucose uptake and lipid metabolism, such as *Glut4* (Fig. 1E), *Fasn*, and *Lpl* (Fig. 1F).

### IL-4 inhibits adipogenesis by affecting cell proliferation at the mitotic clonal expansion phase

**Fig. 2.**
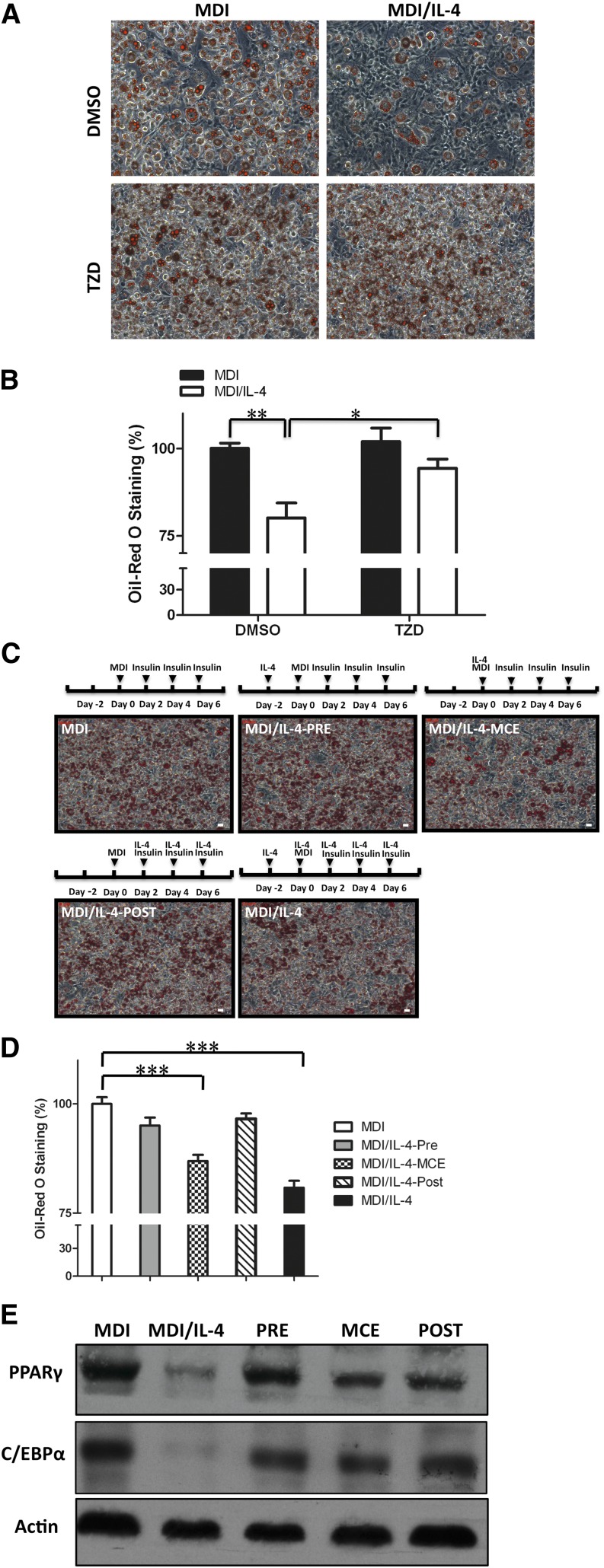
IL-4 inhibits adipogenesis at early MCE stage. Effect of IL-4 inhibitory effect on differentiation under various schemes of IL-4 treatment was analyzed. A, B: PPARγ agonist TZD rescued adipogenesis under IL-4 treatment. 3T3-L1 cells were treated with 10 μM TZD on day 4, and lipid accumulation was examined by ORO staining on day 8. B: The ORO staining in cells was extracted with isopropanol and quantitated at optical density 490 nm. *P < 0.05 DMSO versus TZD; **P < 0.01 MDI versus MDI/IL-4. Data are presented as the mean ± SEM (n = 3). C, D: Schematic illustrator indicates the different IL-4 exposure periods of adipocytes during differentiation (PRE, IL-4 treatment from day −2 to 0; MCE, IL-4 treatment from day 0 to 2; POST, IL-4 treatment from day 2 to 8). C: Intracellular lipid contents were stained by ORO on day 8. Scale bar, 10 μm. D: The corresponding lipid contents of staining results were measured by spectrophotometer. Data are presented as the mean ± SEM (n = 3). ***P < 0.001 compared with MDI. E: Expression of C/EBPγ and PPARγ in adipocytes with differential IL-4 treatment as indicated in (C) was analyzed. Briefly, cells were treated with IL-4 as indicated, and then cell lysates were harvested on day 8 and subjected to Western blotting with β-actin as loading control.

TZD is an adipogenic compound that activates multiple downstream genes in adipocytes through binding to PPARγ (20). To further verify the inhibitory effects of IL-4 toward adipogenesis, we examined whether TZD treatment could rescue the differentiation of IL-4-treated adipocytes. Our results demonstrate that, in the presence of TZD treatment, the degree of lipid accumulation in IL-4-treated cells was comparable with that in control counterparts (**Fig. 2A, B**). This suggests that the inhibitory effect of IL-4 on adipogenesis is targeted at the early stage of differentiation.

The entire adipogenesis process can be divided into the initial mitotic clonal expansion (MCE) and the terminal differentiation phase. When cells are induced to differentiate, they first undergo several rounds of cell cycle to proliferate during the MCE phase, which lasts about 2 days, followed by the differentiation phase through which they acquire the characteristics of mature adipocytes (17, 21). For dissecting the IL-4 inhibitory effect on adipogenesis, cells were induced to differentiate under various schemes of IL-4 treatment as shown in Fig. 2C, and then the extent of adipocyte differentiation was examined. The results showed that while lipid accumulation was significantly suppressed by exposing the cells to IL-4 during the entire differentiation process (MDI/IL-4), short-term IL-4 exposure at the MCE stage (MDI/IL-4-MCE) was sufficient to inhibit adipogenesis (Fig. 2C, D). Parallel to the ORO staining results (Fig. 2C, D), the protein expression of C/EBPα and PPARγ was decreased in MDI/IL-4-MCE and MDI/IL-4 cells (Fig. 2E). The results support our previous observation that IL-4 exerts an inhibitory effect on adipogenesis at the early MCE phase.

**Fig. 3.**
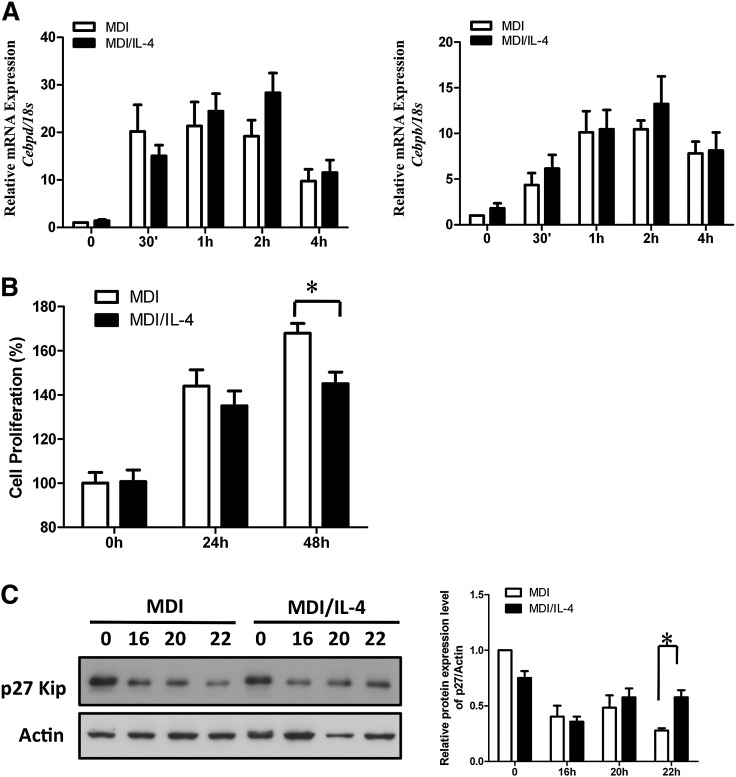
IL-4 partially inhibits cell cycle progression at the MCE stage. Expression levels of transcription factors and cell proliferation activity during the MCE stage under IL-4 treatment were analyzed. A: mRNA expression of C/EBPβ (*Cebpb*) and C/EBPδ (*Cebpd*) in 3T3-L1 cells was analyzed by real-time PCR at the indicated time in the absence or presence of 10 ng/ml IL-4. B: 3T3-L1 cells were induced into differentiation in the absence or presence of IL-4, and then cells were trypsinized, stained with trypan blue, and counted at the indicated time. Data are presented as the mean ± SEM (n = 9). *P < 0.05 MDI/IL-4 versus MDI. C: Expression of p27 Kip was analyzed at the indicated time in the absence or presence of IL-4 treatment by Western blotting with β-actin as loading control. Quantitative results are presented as the mean ± SEM (n = 3). *P < 0.05 compared with MDI.

C/EBPβ and C/EBPδ are important transcription factors which are upregulated at the MCE phase and subsequently activate PPARγ and C/EBPα to trigger adipogenesis. Thus, the putative regulation of IL-4 to the expression of C/EBPβ and C/EBPδ, as well as cell proliferation capacity, were subsequently analyzed by qPCR and trypan blue exclusion, respectively. The results showed that mRNA expression patterns of *Cebpb* and *Cebpd* were not affected (**Fig. 3A**). However, cell numbers in the MCE phase were significantly decreased by about 20% under IL-4 treatment (Fig. 3B), which was parallel to the degree of IL-4 reduced lipids accumulation (Fig. 1A, B). Expression of p27 Kip, the key regulator of cell cycle in the MCE stage, was subsequently examined to verify the above results (22). While p27 Kip expression declined in MDI and MDI/IL-4 once the cells were induced to differentiate, its level in MDI/IL-4 cells was significantly higher than in MDI cells at 22 h postinduction (Fig. 3C). These data suggest that IL-4 inhibits adipocyte differentiation by regulating cell proliferation at the MCE phase.

### IL-4 inhibits adipogenesis through the STAT6 pathway

**Fig. 4.**
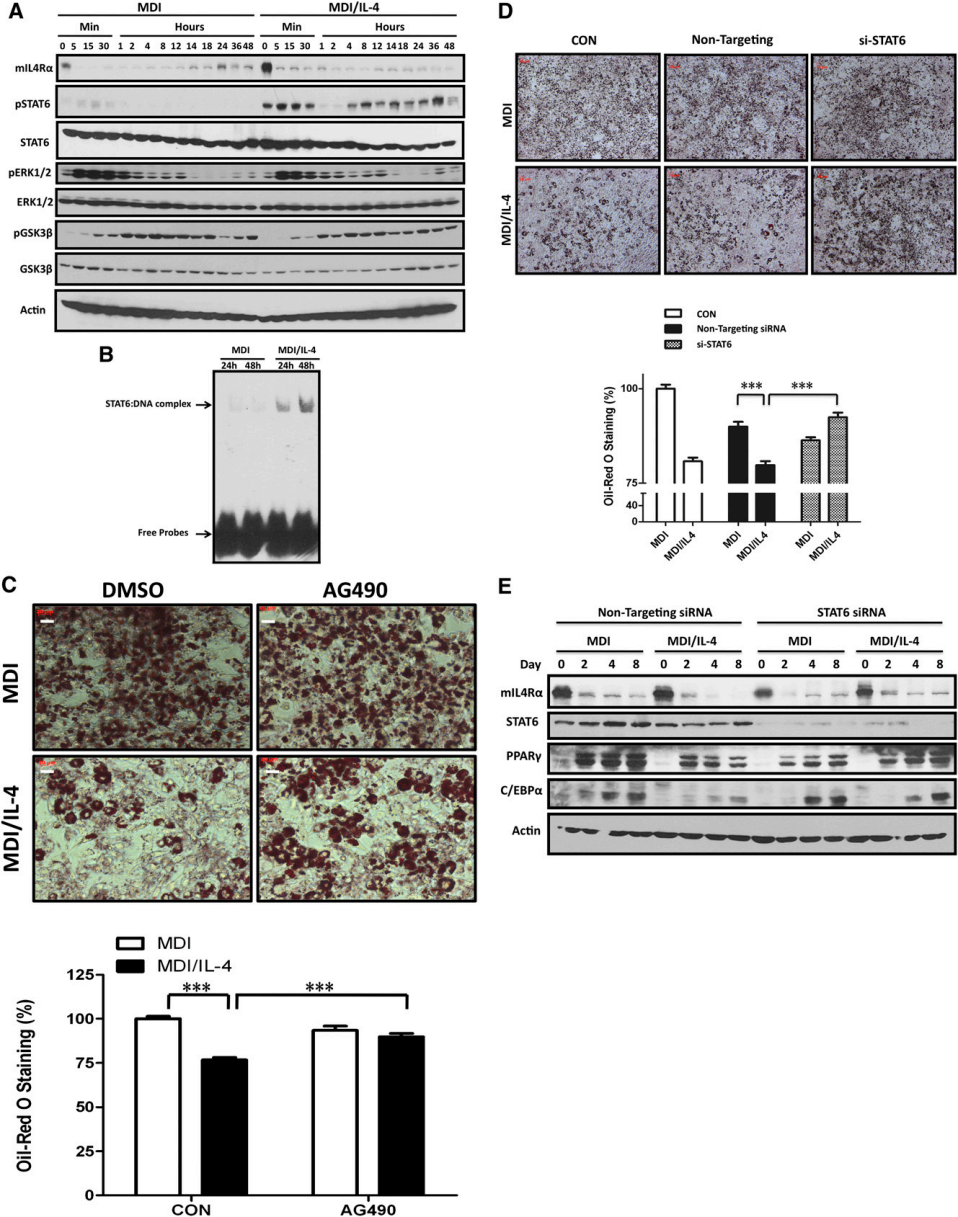
IL-4 induces STAT6 signaling pathway during adipogenesis. Expression and phosphorylation levels of molecules in the signaling pathway at the MCE stage under IL-4 treatment were analyzed. A: 3T3-L1 cells were induced to differentiate with MDI cocktail in the absence or presence of 10 ng/ml IL-4. Cell lysates were harvested at the indicated times and then subjected to Western blotting analysis using antibodies against STAT6, phospho-STAT6 (Tyr^641^), phospho-ERK1/2, total ERK1/2, pGSK3β, and total GSK3β. B: Cells were allowed to differentiate in the absence or presence of 10 ng/ml IL-4. The nuclear extracts were harvested at the indicated times and then subjected to EMSA experiments. The upper arrow indicates the STAT6-probe complex. The lower arrow indicates the unbound free probe. C: Pretreatment with AG490 (10 μM) rescued IL-4-inhibited adipogenesis. 3T3-L1 cells were pretreated with 10 μM AG490 and then induced to differentiate in the absence or presence of IL-4. Lipid accumulation was observed by ORO staining and quantified. Scale bar, 20 μm. ***P < 0.001. D: 3T3-L1 cells were transfected with 100 nM STAT6 siRNA for 24 h and then subjected to differentiation in the absence or presence of 10 ng/ml IL-4. Lipid accumulation was observed by ORO staining on day 8 and quantified. Scale bar, 10 μm. ***P < 0.001. E: Expression of PPARγ and C/EBPα in 3T3-L1 cells transfected with 100 nM nonspecific siRNA or STAT6 siRNA. Data are presented as the mean ± SEM (n = 9). CON, control.

We next investigated the signaling pathways responsible for the inhibition of adipogenesis by IL-4. As shown in **Fig. 4A**, while phosphorylation of glycogen synthase kinase 3β (GSK3β) was not altered by IL-4, activation of extracellular signal-regulated kinases 1/2 (ERK1/2) was partially suppressed.

STAT6, an important downstream molecule in IL-4 signaling, is abundantly expressed in 3T3-L1 cells (23, 24). Therefore, we investigated to determine whether IL-4 would inhibit the MCE phase through STAT6. Our data indicate that IL-4 significantly induces STAT6 phosphorylation, which persisted to 48 h after induction (Fig. 4A). Notably, IL-4 induced the binding of STAT6 to its target sequences, although IL-4Rα expression was rapidly downregulated after induction (Fig. 4A, B). These data demonstrate that IL-4 may inhibit adipogenesis through STAT6-dependent signaling, bypassing IL-4R during the early MCE phase of adipogenesis.

To verify the above observation, cells were treated with Janus kinase inhibitor AG490 in the presence or absence of IL-4 during differentiation, and degrees of adipogenesis were determined. The results indicated that the inhibitory effects of IL-4 on adipocyte differentiation were blocked by AG490 (Fig. 4C). We next investigated to determine whether the IL-4-mediated inhibition of lipid accumulation could be sequestered when STAT6 expression was silenced. As shown in Fig. 4D, STAT6 siRNA specifically counteracted the IL-4-inhibited effects on adipogenesis. Moreover, the expression levels of PPARγ and C/EBPα were recovered in STAT6 siRNA-treated MDI/IL-4 cells (Fig. 4E). These data strongly suggest that IL-4 inhibits the expression of PPARγ and C/EBPα through the STAT6 pathway, which results in the inhibition of adipogenesis.

### IL-4 enhances basal and ISO-induced lipolysis in mature adipocytes

**Fig. 5.**
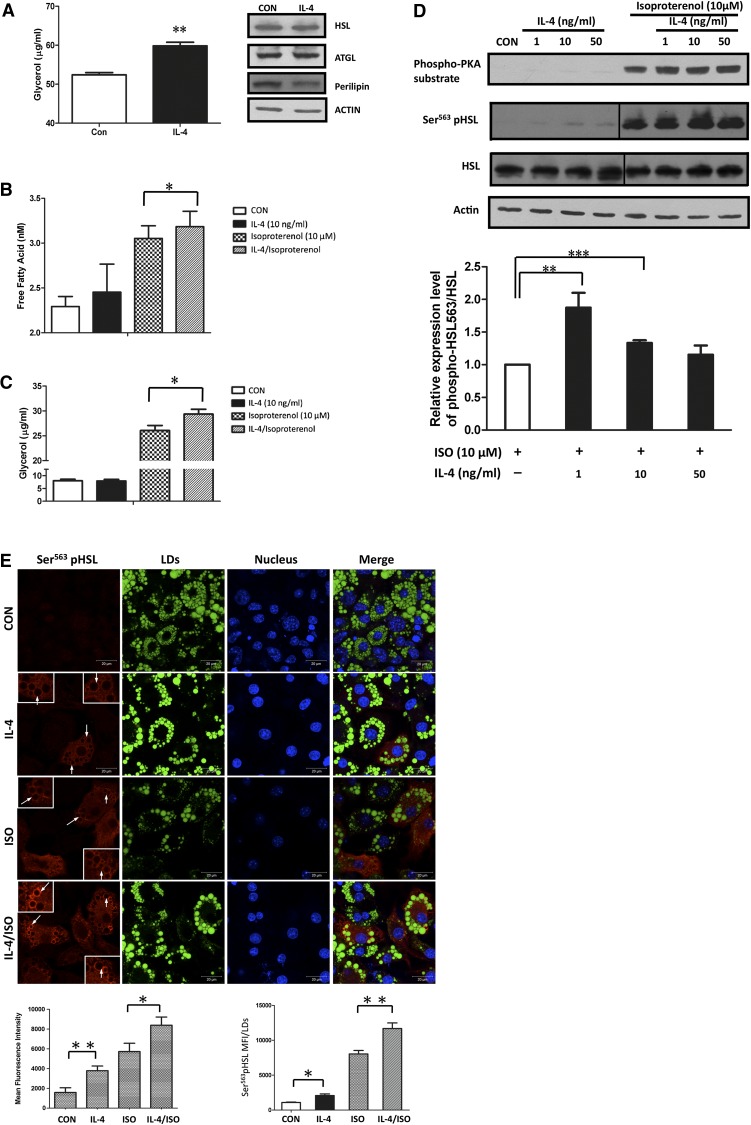
IL-4 enhances basal and ISO-stimulated lipolysis in mature adipocytes. Effects of IL-4 on lipolysis and HSL expression/activity in 3T3-L1 mature adipocytes were analyzed. A: Left panel: Cells were treated with 10 ng/ml IL-4 for 24 h. Culture medium was collected and glycerol content was determined. Data are presented as the mean ± SEM (n = 8). **P < 0.01 compared with untreated control. Right panel: HSL, ATGL, and perilipin protein levels in cells after 24 h of 10 ng/ml IL-4 treatment were analyzed by Western blotting with β-actin as a loading control. B, C: Cells were treated with 10 ng/ml IL-4 for 3 h followed by 10 μM ISO for 1 h. Culture medium was collected and glycerol and NEFA contents were determined. Data are presented as the mean ± SEM (n = 8). **P < 0.01. D: Upper panel: cells were treated with IL-4 in the absence or presence of ISO. Cell lysates were harvested and subjected to Western blotting analysis using antibodies against phospho-PKA substrate, Ser^563^-pHSL, and total HSL with β-actin as a loading control. Lower panel: quantitative results are presented as the mean ± SEM (n = 3). **P < 0.01 and ***P < 0.001. E: Ser^563^-pHSL was recruited to the LD surface (arrows). Cells were treated with vehicle (DMSO), 10 ng/ml IL-4, 10 μM ISO, or 10 μM ISO with 10 ng/ml IL-4 for 1 h prior to fixation. Cells were immunolabeled with Ser^563^-pHSL (red), LDs (BODIPY, green), and DAPI (blue). Scale bar, 20 μm. Mean fluorescence intensity (MFI) of the cells and LDs were analyzed using ZEN 2009 software. Data are presented as mean ± SEM of three separate experiments. *P < 0.05 and **P < 0.01.

Our previous study showed that IL-4-treated mice have higher serum nonesterified fatty acid (NEFA) levels and fewer fat pads (16). We speculated that IL-4-induced NEFA elevation might result from its capacity to inhibit lipid accumulation while promoting lipolysis in adipose tissues. In this context, we examined the effect of IL-4 on lipolysis in mature adipocytes to verify the above hypothesis. As anticipated, glycerol levels were significantly increased by IL-4, which suggests that IL-4 has pro-lipolytic activity (**Fig. 5A**). Subsequently, we analyzed whether IL-4 induced lipolysis by upregulating the expression and/or activities of ATGL and HSL, the key enzymes for triacylglycerol hydrolysis and NEFA release (25). Our results showed that neither the expression of ATGL and HSL (Fig. 5A) nor the mRNA levels of CGI-58 (data not shown), the regulator of ATGL lipolytic activity (26), was altered by IL-4. We further investigated whether IL-4 had a synergistic effect with the lipolysis-inducing agent, ISO, to enhance lipolysis. Levels of both glycerol and NEFA were significantly increased by combined ISO and IL-4 treatment, compared with the counterparts in the environment containing either ISO or IL-4 (Fig. 5B, C). The results suggest that IL-4 harbors the capacity to promote lipolysis both at the basal level and the ISO-stimulated status.

HSL lipolytic activity is primarily controlled by phosphorylation status at Ser^563^ (27). Therefore, the amounts of Ser^563^-phosphorylated (p)HSL were examined in the presence of IL-4 and/or ISO treatment. Our results showed that, while the Ser^563^-pHSL level was slightly elevated (∼1.5- to 2-fold) by IL-4, it was synergistically increased under ISO-induced lipolysis in the presence of IL-4 (Fig. 5D). Whereas, neither IL-4 nor combined treatment affected HSL protein levels. In addition to being phosphorylated, the translocation of HSL from cytosol to the surface of lipid droplets (LDs) is required for it to gain access to the lipid core and exert lipolytic activity. Accordingly, the localization of pHSL under IL-4 or IL-4/ISO treatment was analyzed. Ser^563^-pHSL was ubiquitously detected in cytosol under IL-4 treatment, whereas, IL-4 synergistically enhanced the translocation of Ser^563^-pHSL from cytosol to the surface of the LDs under ISO-stimulated lipolysis (Fig. 5E). Collectively, these data suggest that IL-4 alone stimulates lipolysis by increasing HSL activity. In addition, IL-4 shows synergistic effects on promoting HSL phosphorylation and translocation with pro-lipolytic signals such as ISO.

### IL-4 promotes lipolysis through the PKA pathway in mature adipocytes

**Fig. 6.**
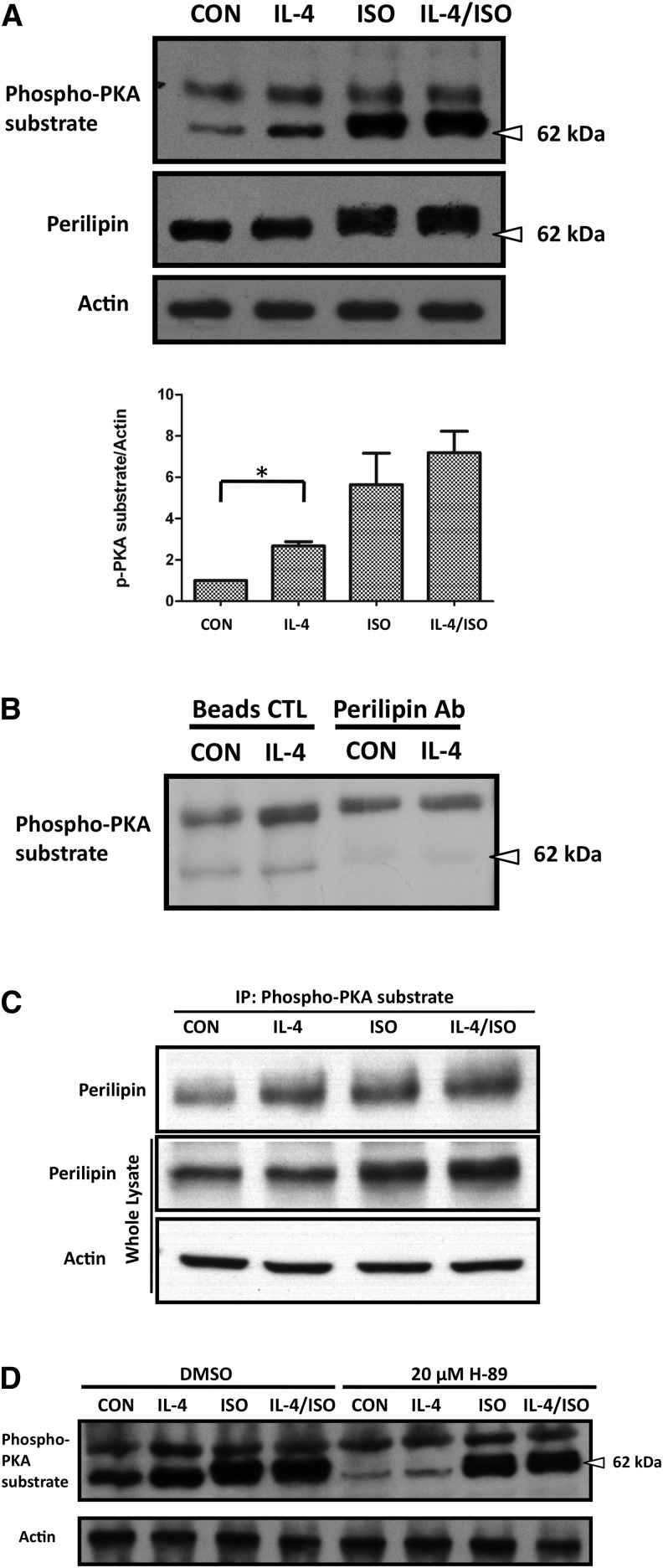
IL-4 promotes perilipin phophorylation in mature adipocytes via the PKA pathway. Expression and phosphorylation levels of perilipin in 3T3-L1 mature adipocytes under IL-4 treatment were analyzed. A: Cells were treated either with 10 ng/ml IL-4 or combined with 10 μM ISO treatment for 1 h. Cell lysates were harvested and subjected to Western blotting analysis using antibodies against phospho-PKA substrate and perilipin with β-actin as a loading control. Data are presented as mean ± SEM of three separate experiments. *P < 0.05. B, C: The 62 kDa phospho-PKA substrate was identified as perilipin. B: Cells were treated with 10 ng/ml IL-4 for 1 h. Perilipin in the cell extracts was first depleted by anti-perilipin antibodies, and then the perilipin-cleared supernatant was harvested and subjected to Western blot analysis. C: Cell lysates after IL-4 treatment were immunoprecipitated with anti-phosho-PKA substrate antibody and subjected to Western blot analysis. D: Cells were pretreated with 20 μM PKA inhibitor H-89 for 1 h and followed by IL-4 treatment in the absence or presence of 10 μM ISO for 1 h. Cell lysates were harvested and subjected to Western blotting analysis using antibodies against phospho-PKA substrate with β-actin as a loading control.

PKA is an upstream regulator that promotes HSL activity by enhancing HSL phosphorylation at Ser^563^ (28). Accordingly, it was tempting for us to assess whether IL-4 induces lipolysis by upregulating HSL activity through PKA. As shown in Fig. 5D and **Fig. 6A**, the level of phosphorylated PKA substrate was increased in both the IL-4 and IL-4/ISO treatment, which supports our speculation that IL-4 enhances HSL activity through the PKA pathway. Notably and interestingly, a prominent ISO-dependent 62 kDa phospho-PKA substrate was detected (Fig. 6A). In addition, phosphorylated perilipin levels were also increased by IL-4 (Fig. 6A). We suspected that perilipin was a potential candidate for the detected 62 kDa phospho-PKA substrate, as it is the major phosphorylated protein in adipocytes responding to increased cAMP concentration (29, 30).

To test the above speculation, perilipin was first immunoprecipitated from cell lysates and the perilipin-depleted supernatant was blotted with the phospho-PKA substrate antibody. Figure 6B shows that the 62 kDa protein was no longer detected in the perilipin-depleted supernatant, supporting the assumption that it was perilipin. In addition, the immunoprecipitation-immunoblotting results also demonstrated that IL-4 enhances perilipin phosphorylation by PKA (Fig. 6C). We further analyzed the phosphorylated perilipin levels by treating the PKA inhibitor, H-89, in adipocytes exposed to either IL-4 or IL-4/ISO to verify whether IL-4-stimulated lipolysis is mediated by activating cAMP-dependent PKA. Our results showed that H-89 abrogated IL-4-induced PKA-dependent perilipin phosphorylation (Fig. 6D). These data support our speculation that IL-4 promotes lipolysis by enhancing phosphorylation of HSL and perilipin through the PKA pathway.

## DISCUSSION

Pro-inflammatory cytokines have been shown to be involved in the T2DM etiology. However, little is known about the role of other cytokines in diabetic pathogenesis (31). In our previous studies, a significant association between IL-4 promoter polymorphisms and T2DM was identified (14), as well as that between IL-4R and HDL-C (15). We further demonstrated that IL-4 regulates glucose and lipid metabolism by promoting insulin sensitivity and glucose tolerance, and inhibiting lipid deposits (16). In the present study, our results reveal that, in addition to inhibiting adipocyte differentiation through the STAT6 pathway, IL-4 also enhances lipolysis by upregulating perilipin phosphorylation and HSL activity/translocation through the PKA pathway. These observations are consistent with our previous findings and reveal that IL-4 inhibits lipid deposits. To the best of our knowledge, this is the first study that demonstrates the novel roles of IL-4 in regulating lipid metabolism through inhibiting adipogenesis and promoting lipolysis.

The adipogenic programming is finely orchestrated and driven by the sequential activation of transcription factors such as C/EBPβ, C/EBPδ, PPARγ, and C/EBPα, which trigger the cells to enter the terminal differentiation by controlling the coordinate expression of a number of key adipocyte genes. Our results reveal that while IL-4 downregulates the expression of C/EBPα and PPARγ, C/EBPβ and C/EBPδ are not affected (Figs. 1B, 3A). In addition, IL-4 inhibits adipogenesis by targeting at the MCE phase (Fig. 3C). IL-4 is known to inhibit cell cycle progression through STAT6, which alters the expression of several key regulators such as p21(Waf1) and p27(Kip) (32–36). We speculate that the net anti-adipogenic effect of IL-4 might result from its capacity to either elevate the expression of p27/p21 or protect these proteins from proteasome degradation through the STAT6 pathway, and thus inhibit MCE during adipogenesis. The result that the expression of p27 in IL-4-treated cells is higher than that in control counterparts (Fig. 3C) further supports the above speculation.

The rapidly elevated C/EBPβ/δ at the early stage of adipogenesis remains inactive until it is phosphorlyated (20, 21). Only after being phosphorylated can C/EBPβ acquire DNA-binding and *trans*-activating activity to make preadipocytes traverse the G1-S checkpoint at the MCE phase (37, 38). C/EBPβ is phosphorylated sequentially, first by MAPK and then much later by GSK3β (37, 38). MAPK-mediated phosphorylation of C/EBPβ Thr^188^ occurs ∼4 h after induction of differentiation, and thus is required for MCE, C/EBPβ DNA binding activity, and terminal differentiation (37, 38). Our results show that activation of MAPK p44/42 (ERK1/2) is partially suppressed by IL-4 (Fig. 4A), suggesting that Thr^188^ phosphorylation and, therefore, C/EBPβ DNA binding activity might also be partially inhibited. However, this speculation awaits further investigation.

The role of STATs in adipocyte differentiation has been extensively studied, including STAT1, -3, and -5 (39–44). However, little is known about the role of STAT6 in adipogenesis. We demonstrate that IL-4 inhibits adipogenesis by inducing STAT6 activation in 3T3-L1 cells, although IL-4Rα is rapidly downregulated at the early phase of differentiation (Fig. 4A). The result that STAT6 knockdown rescues IL-4-inhibited adipogenesis further verifies the above observation. Interestingly, IL-4 transgenic mice show a considerably reduced adipocyte layer in the dermis (45), suggesting that IL-4 inhibits lipid storage and adipocyte differentiation. Our previous animal study also indicates that IL-4 administration leads to lower fat mass and reduced lipid contents in adipocytes (16). Our observations echo the recent finding from Ricardo-Gonzalez et al. (46), that STAT6-null mice gain significantly less weight and have smaller WAT depots when challenged with a high-fat diet. Taken together, this evidence supports the premise that IL-4-inhibited adipogenesis is a STAT6-dependent effect.

Nevertheless, the finding that IL-4 suppresses adipocyte differentiation by mediating PPARγ expression is contradictory to another study (47). We suggest that this discrepancy may result from different experimental conditions. In our study, preadipocytes were maintained in medium containing calf serum, which was replaced by FBS when the cells were induced to differentiate. In addition, the MDI cocktail in the induction medium was removed after 48 h and replaced by insulin-containing fresh medium. Whereas, the Hua, Deng, and Harp (47) study used FBS-containing medium for both preadipocyte maintenance and the entire adipocyte differentiation process, and the cells were exposed to MDI cocktail for 72 h. Therefore, the possibility that differential gene expression patterns of 3T3-L1 cells responding to different experimental conditions cannot be ruled out.

The cAMP-dependent PKA pathway is the major route leading to lipolysis, through which adenyl cyclase is activated by the stimulated Gs-coupled receptors (48). The subsequently increased intracellular cAMP levels then result in PKA activation, followed by HSL phosphorylation, translocation, and access to LDs for mediating triacylglycerol hydrolysis (48). In the present study, our results show that IL-4 enhances the major phosphorylation site of HSL, Ser^563^, by PKA (Fig. 5D). Moreover, IL-4 increases the level of PKA-dependent perilipin phosphorylation, which is attenuated by the PKA-specific inhibitor H-89 (Fig. 6C). These results strongly suggest that IL-4 participates in lipid metabolism by evoking a conventional lipolytic pathway in adipocytes. Unlike TNF-α and IL-6, which are known to stimulate lipolysis by activating the ERK1/2 pathway and suppressing the expression of multiple genes involved in preventing lipolysis (49, 50), IL-4 does not alter the expression of HSL, perilipin, ATGL (Fig. 5A), and CGI-58 or activate ERK signaling (data not shown). How IL-4 transverses its anti-adipogenesis and pro-lipolysis signaling needs further investigation because expression of IL-4Rα rapidly declines when the cells enter differentiation (Fig. 4A)

In addition to inhibiting adipocyte differentiation, our results also show that IL-4 modulates the expression of adiponectin and resistin (Fig. 1F). Resistin is an adipocyte-secreted hormone which has been indicated in promoting lipolysis, glucose intolerance, and insulin resistance (51). The downregulated resistin in IL-4-treated cells supports our previous conclusion that IL-4 is involved in lipid metabolism by inhibiting lipid accumulation in fat tissues, which leads to decreased weight gain and fat mass in IL-4-treated mice (16). On the contrary, discrepancy in adiponection levels was observed in the present study. Adiponectin is known to have anti-lipolytic ability (52). We hypothesize that the increased adiponectin levels in IL-4-treated mice should reflect the integrative effects of IL-4 on metabolism in the complicated interplay/network of IL-4 and other intrinsic physiological factor(s) (16). Hence, we suggest that regulating adipokine secretion may be one of the potential mechanisms by which IL-4 modulates energy metabolism. Nevertheless, as a lipodystrophic state in mice would perhaps lead to ectopic lipid deposition and insulin resistance (53), whether reduced adipogenesis and fat storage resulting from IL-4 administration would lead to the above metabolic abnormality in certain organs needs further investigation.

Obesity is accompanied by an increased infiltration of adipose tissue macrophages (ATMs), possibly recruited by the signals released from damaged or dead hypertrophic adipocytes (54). While the ATMs in lean animals correspond to the M2 anti-inflammatory phenotype, the ATMs from obese animals are predominately the M1 pro-inflammatory phenotype (55). It is known that adipocytes produce IL-4 and IL-13 cytokines locally, establishing a reciprocal functional crosstalk between adipocytes and the resident M2 ATMs, which leads to suppression of M1 macrophages as well as the improvement of lipid metabolism and insulin sensitivity (12, 13). Treatment of adipocytes with conditioned medium from M2 macrophages also induces phosphorylation of perilipin and HSL (56). Therefore, except for the results demonstrating IL-4 directly inhibits adipogenesis and promotes lipolysis through the STAT6 and the PKA pathway, respectively, we infer that the microenvironmental IL-4 may promote M2 macrophage polarization, which plays positive roles in lipid metabolism by reducing inflammation in adipose tissues and therefore preventing the incidence of insulin resistance.

The combined evidence from the present study and our previous reports (14–16) uncovers the novel role of IL-4 in regulating lipid metabolism by the following findings. First of all, significant associations between IL-4 gentoypes and T2DM (14), as well as between the genotypes of IL-4 and IL-4Rα with HDL-C, are observed (15). Second, IL-4-injected mice show decreased fat mass and increased serum NEFA levels, revealing the inhibitory capacity of IL-4 to lipid accumulation in fat tissue and the subsequent elevated circulatory NEFA levels (16). Third, consistent with our animal study results, the present study indicates that IL-4 harbors anti-lipogenic ability by suppressing adipocyte differentiation and promoting lipolysis in mature adipocytes. Hopefully, the above findings not only provide new insights into the interaction among cytokines, lipid metabolism, and obesity, but also provide clues to the underlying mechanism which leads to the ultimate T2DM metabolic tragedy.
